# Association of Irregular Pigment Epithelial Detachment in Central Serous Chorioretinopathy with Genetic Variants Implicated in Age-related Macular Degeneration

**DOI:** 10.1038/s41598-020-57747-8

**Published:** 2020-01-27

**Authors:** Soo Chang Cho, Na-Kyung Ryoo, Jeeyun Ahn, Se Joon Woo, Kyu Hyung Park

**Affiliations:** 1 Department of Ophthalmology, Seoul National University College of Medicine, Seoul National University Bundang Hospital, Seongnam, Republic of Korea; 2 Department of Ophthalmology, Ewha Womans University Mokdong Hospital, Seoul, Republic of Korea; 3 Department of Ophthalmology, Veterans Health Service Medical Center, Seoul, Republic of Korea; 4 Department of Ophthalmology, Seoul National University College of Medicine, Seoul Metropolitan Government Seoul National University Boramae Medical Center, Seoul, Republic of Korea

**Keywords:** Retinal diseases, Genetics research

## Abstract

We evaluated phenotype and genotype correlation of central serous chorioretinopathy (CSC) patients with or without irregular pigment epithelial detachment (PED) on optical coherence tomography (OCT). For CSC, a flat, irregular protrusion of retinal pigment epithelium (RPE) with hyper-reflective sub-RPE fluid on OCT was defined as an irregular PED. Participants were classified into 5 subgroups; (1) total CSC (n = 280) (2) CSC with irregular PED (n = 126) (3) CSC without irregular PED (n = 154) (4) typical choroidal neovascularization (CNV) (n = 203) and (5) polypoidal choroidal vasculopathy (PCV) (n = 135). Ten known major AMD-associated single-nucleotide polymorphisms (SNPs) were analyzed. Age, sex adjusted logistic regression was performed for the association between subgroups. Association analysis between CSC without irregular PED and CNV revealed that significant difference for rs10490924 in *ARMS2*, rs10737680 in *CFH*, and marginally significant difference for rs800292 in *CFH*. Between CSC without irregular PED and PCV, rs10490924, rs10737680, and rs800292 were significantly different. In contrast, CSC with irregular PED and CNV revealed no SNP showing significant difference. Between CSC with irregular PED and PCV, only rs10490924 was significantly different. CSC with irregular PED and CSC without irregular PED revealed significant difference for rs800292, and marginal difference for rs10737680. These findings suggest CSC patients with irregular PED are genetically different from those without irregular PED and may have genetic and pathophysiologic overlap with AMD patients.

## Introduction

Central serous chorioretinopathy (CSC) is a generally self-limiting disease characterized by localized serous detachment of central retina with retinal pigment epithelial abnormality. Recent advance of imaging devices like optical coherence tomography (OCT) showed serous retinal detachment frequently combined with retinal pigment epithelial detachment (PED) with underlying thickened choroid and choroidal hyperpermeability. CSC is also known as one of the pachychoroid spectrum disease due to its thick choroid.

Pachychoroid features include focal or diffuse increase in choroidal thickness, choroidal hyperpermeability, and dilated choroidal vessels^1,2^. Pachychoroid spectrum diseases refer to a group of clinical entities that have a common feature of pachychoroid and share an underlying disease mechanism. Pachychoroid spectrum diseases include pachychoroid pigment epitheliopathy (PPE), CSC, pachychoroid neovasculopathy (PNV), and polypoidal choroidal vasculopathy (PCV)^3^. In this spectrum, PNV has been postulated as a possible precursor of PCV^4^.

If these diseases are in the same spectrum of disease, they might share similar genetic architecture. Previously, some report showed CSC is related with age-related macular degeneration (AMD) risk genotype but the direction is apposite^5–8^. We also performed genotypic comparison study on the patients with CSC, PCV and control. However, we could not find any genetic association between CSC and PCV^9^.

PED is a well-known feature of CSC. It occurs in 70–100% of cases with CSC^10^. With progress of optical coherence tomographic imaging, PED in CSC showed diverse size, shape and nature like small RPE bump like elevation, large dome shape or regular PED and sometimes flat, irregular PED.

Some of CSC patients with irregular PED showed vascular de-correlation signal by OCT angiography (OCTA) which suggests hidden sessile choroidal neovascularization (CNV) present in the irregular PED. There have been some studies revealing that irregular PED is associated with the presence of CNV in eyes with chronic CSC^11–17^. Due to diverse features of OCT, OCTA, and genetic studies, we presumed that CSC is not a single disease but may be mixed disease of similar phenotype. Because of the mixed nature of the disease, CSC is vaguely classified as acute, chronic or atypical CSC without established concrete definition. This is also a good reason why CSC has diverse clinical features and different visual prognosis.

Considering previous studies on the association of the irregular PED in CSC and the CNV or pachychoroid spectrum disease, irregular PED in CSC can be regarded as a hidden CNV in CSC or early stage of PNV.

Consequently, we speculate that not the whole CSC patients, but CSC patients with irregular shaped PED may have similar genetic risk factors those associated with PCV or neovascular AMD. There has been no study on genetic difference between CSC patients with irregular PED and those without irregular PED. To find out clinical implications of irregular PED in CSC, we investigate whether CSC patients with irregular PED have different genotype compared to those without irregular PED and also analyze the genetic association between the CSC patients with irregular PED and the patients with PCV or neovascular AMD.

## Results

### Characteristics of patients

The demographic details of the study population are shown in Table 1. Proportion of the female was 28.9% for total CSC, 46.3% for CNV, and 28.9% for PCV (*P* < 0.001). The proportion of the female in group of CSC with irregular PED was higher than group of CSC without irregular PED (34.9%, 24.0%, respectively; *P* = 0.045). The mean age (±standard deviation [SD]) was 48.1 (±8.1) years in total CSC, 72.3 (±8.4) years in CNV, and 67.7 (±7.5) years in PCV (*P* < 0.001). The mean age was higher in group of irregular PED (+) than irregular PED (−) in CSC (50.2 ± 8.0 years, 46.4 ± 7.9 years; *P* < 0.001). The mean subfoveal choroidal thickness (SFCT) (±SD) was 397.9 (±97.7) *μ*m in total CSC, 217.1 (±76.6) *μ*m in CNV, and 269.0 (±94.6) *μ*m in PCV (*P* < 0.001). The mean SFCT was slightly thinner in group of irregular PED (+) than irregular PED (−) in CSC (390.7 ± 104.3*μ*m, 403.8 ± 91.9*μ*m; *P* = 0.266).

**Table 1:** Demographics of the Study Population.

Characteristics	Total CSC	Irregular PED (+) in CSC	Irregular PED (−) in CSC	CNV	PCV	P-value for total patients	P-value for CSC only
No. of subjects	280	126	154	203	135	NA	NA
Sex (male: female)	199 (71.1%): 81 (28.9%)	82 (65.1%): 44 (34.9%)	117 (76.0%): 37 (24.0%)	109 (53.7%): 94 (46.3%)	96 (71.1%): 39 (28.9%)	<0.001*	0.045**
Mean age ± SD (yrs)	48.1 ± 8.1	50.2 ± 8.0	46.4 ± 7.9	72.3 ± 8.4	67.7 ± 7.5	<0.001^†^	<0.001^‡^
Age range (yrs)	28–74	32–74	28–69	51–92	50–88	NA	NA
Subfoveal choroidal thickness (*μ*m)	397.9 ± 97.7	390.7 ± 104.3	403.8 ± 91.9	217.1 ± 76.6	269.0 ± 94.6	<0.001^†^	0.266^‡^

CSC = central serous chorioretinopathy; PED = pigment epithelial detachment; CNV = choroidal neovascularization;

PCV = polypoidal choroidal vasculopathy; NA = Not appilicable; SD = standard deviation.

*Statistical analysis with Chi-square test among ‘Total CSC’ group, ‘CNV’ group, and ‘PCV’ group.

^†^Statistical analysis with One-way ANOVA test among ‘Total CSC’ group, ‘CNV’ group, and ‘PCV’ group.

**Statistical analysis with Chi-square test between ‘CSC with irregular PED’ group and ‘CSC without irregular PED’ group.

^‡^Statistical analysis with t-test between ‘CSC with irregular PED’ group and ‘CSC without irregular PED’ group.

### Association analysis of 10 known major AMD-associated SNPs between each pair of subgroups

Among 10 SNPs that were analyzed, rs800292 in *CFH* was significantly different between CSC with irregular PED and CSC without irregular PED (*P* = 2.00 * 10^−3^, odds ratio [OR] = 0.57). Rs10737680 in *CFH* showed marginally significant difference between the two groups (*P* = 5.72 * 10^−3^, OR = 0.61) (Table 2).

**Table 2:** Analysis of 10 Age-Related Macular Degeneration Loci in ‘Irregular Pigment Epithelial Detachment (+) of Central Serous Chorioretinopathy’ and ‘Irregular Pigment Epithelial Detachment (−) of Central Serous Chorioretinopathy’ group.

Case	Control	SNP	Gene	Allele	Minor Allele Frequency		Allelic association		Age, sex adjusted analysis	
					case	control	p-value	Odds Ratio (95% CI)	p-value	Odds Ratio (95% CI)
Irregular PED (+) in CSC (N = 126)	Irregular PED (−) in CSC (N = 154)	rs800292	*CFH*	G > A	0.3889	0.526	**0.001212**	0.574 (0.409–0.804)	**0.002001**	0.569 (0.397–0.813)
		rs1061170	*CFH*	T > C	0.1071	0.0649	0.07312	1.728 (0.945–3.161)	0.09028	1.689 (0.921–3.099)
		rs10737680	*CFH*	A > C	0.4365	0.5519	*0*.*006565*	0.629 (0.450–0.879)	*0*.*005722*	0.611 (0.431–0.867)
		rs6795735	*ADAMTS9*	T > C	0.2183	0.1591	0.07328	1.476 (0.963–2.262)	0.1343	1.379 (0.905–2.100)
		rs4698775	*CFI*	T > G	0.2302	0.1818	0.1575	1.345 (0.891–2.032)	0.1396	1.372 (0.902–2.086)
		rs429608	*C2-CFB*	G > A	0.06349	0.1071	0.06896	0.565 (0.303–1.052)	0.08527	0.578 (0.310–1.079)
		rs943080	*VEGFA*	T > C	0.2778	0.3279	0.1999	0.788 (0.548–1.135)	0.1484	0.749 (0.506–1.108)
		rs334353	*TGFBR1*	T > G	0.4802	0.4578	0.5977	1.094 (0.784–1.527)	0.5541	1.111 (0.784–1.575)
		rs10490924	*ARMS2-HTRA1*	G > T	0.4167	0.3214	0.01981	1.508 (1.067–2.132)	0.03382	1.495 (1.031–2.168)
		rs3764261	*CETP*	G > T	0.1746	0.1623	0.6992	1.092 (0.700–1.702)	0.8668	1.042 (0.646–1.681)

Bold character indicates statistically significance (P < 0.005, Bonferroni correction).

Italic character indicates marginal significance.

In the association analysis between total CSC (n = 280) and typical CNV (n = 203), rs10490924 in *ARMS2-HTRA1*, rs10737680 in *CFH* showed marginally significant difference (*P* = 6.05 * 10^−3^, OR = 0.48; *P* = 7.43 * 10^−3^, OR = 2.04, respectively; Supplementary Table 1 in Supplementary Information). Association analysis between total CSC (n = 280) and PCV (n = 135) revealed that significant difference for rs10490924 in *ARMS2-HTRA1* (*P* = 2.10 * 10^−5^, OR = 0.29), rs10737680 in *CFH* (*P* = 4.20 * 10^−3^, OR = 2.13) and marginally significant difference for rs800292 in *CFH* (*P* = 5.59 * 10^−3^, OR = 2.19; Supplementary Table 2 in Supplementary information).

In the association analysis between CSC with irregular PED (n = 126) and typical CNV (n = 203), there was no SNP showing significant difference (Table 3). Association analysis between CSC with irregular PED (n = 126) and PCV (n = 135) revealed significant difference in only one SNP among 10 SNPs (rs10490924 in *ARMS2-HTRA1*; *P* = 4.17 * 10^−3^, OR = 0.41; Supplementary Table 3 in Supplementary information).

**Table 3:** Analysis of 10 Age-Related Macular Degeneration Loci in ‘Irregular Pigment Epithelial Detachment (+) of Central Serous Chorioretinopathy’ and ‘Typical Choroidal Neovascularization’ group.

Case	Control	SNP	Gene	Allele	Minor Allele Frequency		Allelic association		Age, sex adjusted analysis	
					case	control	p-value	Odds Ratio (95% CI)	p-value	Odds Ratio (95% CI)
Irregular PED (+) in CSC (N = 126)	CNV (N = 203)	rs800292	*CFH*	G > A	0.3889	0.2734	**0.001979**	1.691 (1.211–2.363)	0.2083	1.482 (0.803–2.736)
		rs1061170	*CFH*	T > C	0.1071	0.1238	0.5201	0.850 (0.517–1.397)	0.3912	0.683 (0.286–1.632)
		rs10737680	*CFH*	A > C	0.4365	0.33	*0*.*006122*	1.573 (1.137–2.176)	0.1454	1.546 (0.860–2.779)
		rs6795735	*ADAMTS9*	T > C	0.2183	0.1946	0.4636	1.156 (0.785–1.702)	0.9795	1.009 (0.519–1.960)
		rs4698775	*CFI*	T > G	0.2302	0.2069	0.4807	1.146 (0.785–1.674)	0.1986	0.628 (0.309–1.276)
		rs429608	*C2-CFB*	G > A	0.06349	0.06404	0.9777	0.991 (0.521–1.886)	0.4173	0.632 (0.208–1.918)
		rs943080	*VEGFA*	T > C	0.2778	0.2602	0.6228	1.094 (0.766–1.561)	0.1743	1.628 (0.806–3.289)
		rs334353	*TGFBR1*	T > G	0.4802	0.453	0.497	1.115 (0.814–1.529)	0.1383	1.604 (0.859–2.995)
		rs10490924	*ARMS2-HTRA1*	G > T	0.4167	0.6725	**1.26E-10**	0.348 (0.251–0.482)	0.1876	0.679 (0.382–1.208)
		rs3764261	*CETP*	G > T	0.1746	0.1626	0.6874	1.090 (0.717–1.656)	0.4723	0.750 (0.343–1.643)

Bold character indicates statistically significance (P < 0.005, Bonferroni correction).

Italic character indicates marginal significance.

In the association analysis between CSC without irregular PED (n = 154) and typical CNV (n = 203), rs10490924 in *ARMS2-HTRA1*, rs10737680 in *CFH* were significantly different (*P* = 1.45 * 10^−3^, OR = 0.30; *P* = 3.65 * 10^−3^, OR = 2.87, respectively; Table 4). Rs800292 in *CFH* showed marginally significant difference between the two groups (*P* = 6.12 * 10^−3^, OR = 2.68; Table 4). Association analysis between CSC without irregular PED (n = 154) and PCV (n = 135) revealed that significant difference for rs10490924 in *ARMS2-HTRA1*, rs10737680 and rs800292 in *CFH* (*P* = 8.35 * 10^−6^, OR = 0.14; *P* = 3.16 * 10^−3^, OR = 2.78; *P* = 4.91 * 10^−3^, OR = 2.81, respectively; Supplementary Table 4 in Supplementary information).

**Table 4:** Analysis of 10 Age-Related Macular Degeneration Loci in ‘Irregular Pigment Epithelial Detachment (−) of Central Serous Chorioretinopathy’ and ‘Typical Choroidal Neovascularization’ group.

Case	Control	SNP	Gene	Allele	Minor Allele Frequency		Allelic association		Age, sex adjusted analysis	
					case	control	p-value	Odds Ratio (95% CI)	p-value	Odds Ratio (95% CI)
Irregular PED (−) in CSC (N = 154)	CNV (N = 203)	rs800292	*CFH*	G > A	0.526	0.2734	**6.06E-12**	2.949 (2.157–4.031)	*0*.*006124*	2.680 (1.324–5.422)
		rs1061170	*CFH*	T > C	0.06494	0.1238	*0*.*009002*	0.492 (0.286–0.845)	0.02989	0.249 (0.071–0.873)
		rs10737680	*CFH*	A > C	0.5519	0.33	**3.22E-09**	2.501 (1.841–3.398)	**0.003651**	2.867 (1.409–5.832)
		rs6795735	*ADAMTS9*	T > C	0.1591	0.1946	0.2208	0.783 (0.529–1.159)	0.4486	1.352 (0.620–2.951)
		rs4698775	*CFI*	T > G	0.1818	0.2069	0.4032	0.852 (0.585–1.241)	0.6611	0.828 (0.356–1.925)
		rs429608	*C2-CFB*	G > A	0.1071	0.06404	0.03828	1.754 (1.025–3.000)	0.3825	1.645 (0.539–5.023)
		rs943080	*VEGFA*	T > C	0.3279	0.2602	0.05	1.387 (0.999–1.925)	0.1059	1.852 (0.877–3.911)
		rs334353	*TGFBR1*	T > G	0.4578	0.453	0.8981	1.020 (0.757–1.373)	0.1491	1.623 (0.841–3.132)
		rs10490924	*ARMS2-HTRA1*	G > T	0.3214	0.6725	**1.87E-20**	0.231 (0.168–0.317)	**0.001448**	0.302 (0.144–0.631)
		rs3764261	*CETP*	G > T	0.1623	0.1626	0.9936	0.998 (0.668–1.492)	0.9651	0.979 (0.381–2.519)

Bold character indicates statistically significance (P < 0.005, Bonferroni correction).

Italic character indicates marginal significance.

**Figure 1:**
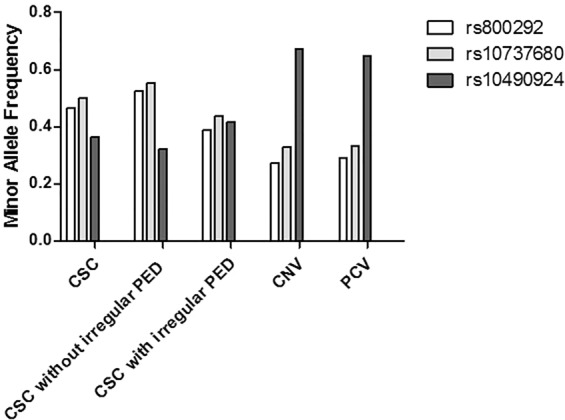
Minor allele frequencies of 3 representative single nucleotide polymorphisms among different groups.

The summary of the above analyses was presented in Table 5 and Fig. 1.

**Table 5:** Summary of the Results: Minor Allele Frequency for Each Single Nucleotide Polymorphism in Each Study Group with Paired Comparions by Age, Sex Adjusted Analysis.

SNP	Gene	Allele	Minor Allele Frequency					Age, sex adjusted analysis p-value Odds Ratio (95% CI)						
			CSC total	CSC IP−	CSC IP+	CNV	PCV	CSC total vs. CNV	CSC total vs PCV	CSC IP− vs. CNV	CSC IP− vs. PCV	CSC IP+ vs. CNV	CSC IP+ vs. PCV	CSC IP+ vs. CSC IP-
rs800292	*CFH*	G > A	0.48	0.54	0.38	0.27	0.29	0.014 1.97 (1.15–3.38)	*0*.*0056* 2.19 (1.26–3.81)	*0*.*0061* 2.68 (1.32–5.42)	**0**.**0049** 2.81 (1.37–5.78)	0.21 1.48 (0.80–2.74)	0.13 1.64 (0.87–3.08)	**0**.**0020** 0.57 (0.40–0.81)
rs1061170	*CFH*	T > C	0.08	0.06	0.11	0.12	0.10	0.13 0.53 (0.23–1.22)	0.21 0.60 (0.27–1.35)	0.030 0.25 (0.07–0.87)	0.066 0.32 (0.09–1.08)	0.39 0.68 (0.29–1.63)	0.58 0.79 (0.33–1.86)	0.090 1.69 (0.92–3.10)
rs10737680	*CFH*	A > C	0.51	0.56	0.43	0.33	0.33	*0*.*0074* 2.04 (1.21–3.43)	**0**.**0042** 2.13 (1.27–3.58)	**0**.**0037** 2.87 (1.41–5.83)	**0**.**0032** 2.78 (1.41–5.49)	0.15 1.55 (0.86–2.78)	0.099 1.63 (0.91–2.90)	*0*.*0057* 0.61 (0.43–0.87)
rs6795735	*ADAMTS9*	T > C	0.19	0.19	0.20	0.19	0.21	0.88 1.05 (0.58–1.90)	0.95 1.02 (0.56–1.85)	0.45 1.35 (0.62–2.95)	0.51 1.31 (0.59–2.91)	0.98 1.01 (0.52–1.96)	0.86 0.95 (0.49–1.86)	0.13 1.38 (0.91–2.10)
rs4698775	*CFI*	T > G	0.22	0.21	0.23	0.21	0.21	0.22 0.67 (0.35–1.27)	0.46 0.80 (0.43–1.47)	0.66 0.83 (0.36–1.93)	0.95 0.97 (0.44–2.17)	0.20 0.63 (0.31–1.28)	0.41 0.75 (0.38–1.48)	0.14 1.37 (0.90–2.09)
rs429608	*C2-CFB*	G > A	0.10	0.12	0.06	0.06	0.06	0.68 1.22 (0.48–3.12)	0.34 1.57 (0.62–3.98)	0.38 1.65 (0.54–5.02)	0.11 2.39 (0.81–7.04)	0.42 0.63 (0.21–1.92)	0.96 0.97 (0.30–3.17)	0.085 0.58 (0.31–1.08)
rs943080	*VEGFA*	T > C	0.29	0.31	0.28	0.26	0.31	0.066 1.77 (0.96–3.26)	0.81 1.07 (0.62–1.85)	0.11 1.85 (0.88–3.91)	0.50 1.26 (0.64–2.51)	0.17 1.63 (0.81–3.29)	0.99 1.01 (0.54–1.89)	0.15 0.75 (0.51–1.11)
rs334353	*TGFBR1*	T > G	0.47	0.47	0.47	0.45	0.40	0.1 1.55 (0.92–2.62)	0.28 1.31 (0.80–2.13)	0.15 1.62 (0.84–3.13)	0.45 1.27 (0.68–2.36)	0.14 1.60 (0.86–3.00)	0.38 1.30 (0.73–2.32)	0.55 1.11 (0.78–1.56)
rs10490924	*ARMS2-HTRA1*	G > T	0.36	0.34	0.39	0.67	0.65	*0*.*0060* 0.48 (0.28–0.81)	**2**.**10E-05** 0.29 (0.16–0.51)	**0**.**0014** 0.30 (0.14–0.63)	**8**.**35E-06** 0.14 (0.06–0.34)	0.19 0.68 (0.38–1.21)	**0**.**0042** 0.41 (0.22–0.76)	0.034 1.50 (1.03–2.17)
rs3764261	*CETP*	G > T	0.16	0.16	0.17	0.16	0.21	0.49 0.78 (0.38–1.59)	0.0078 0.40 (0.21–0.79)	0.97 0.98 (0.38–2.52)	0.063 0.43 (0.18–1.05)	0.47 0.75 (0.34–1.64)	0.017 0.40 (0.19–0.85)	0.87 1.04 (0.65–1.68)

Total CSC (n = 280) CSC IP−: CSC without irregular PED (n = 154) CSC IP+ : CSC with irregular PED (n = 126).

Typical CNV (n = 203) PCV (n = 135).

Bold character indicates statistically significance (P < 0.005, Bonferroni correction).

Italic character indicates marginal significance.

## Discussion

Based on the age, sex adjusted association analysis with 10 AMD-associated SNPs, patients without irregular PED showed significant difference with CNV patients in 2 SNPs, marginally significant difference in 1 SNP, and significant difference with PCV patients in 3 SNPs. In contrast, patients with irregular PED revealed no SNP showing significant difference with CNV patients, and only one SNP showing significant difference with PCV patients. In the association analysis between patients with irregular PED and those without irregular PED, the rs800292 in *CFH* were significantly different, and the rs10737680 in *CFH* showed marginally significant difference. These findings suggest not CSC patients but CSC patients with phenotype of irregular PED may have genetic and pathophysiologic overlap with neovascular AMD patients.

The age, sex adjusted association analysis revealed that patients with irregular PED were genetically different from patients without irregular PED. In regards to genetic profile, patients with irregular PED were closer to patients with CNV or PCV than those without irregular PED. These findings suggest CSC patients with irregular PED may have genetic overlap with neovascular AMD patients. The findings imply that patients with irregular PED may have more tendency to have hidden CNV or to develop into CNV or PCV. This implication is similar to the previous studies that showed irregular PED in CSC or pachychoroid disease appears to be a risk factor of CNV^11,14^. One study using OCTA showed that CNV was detected more in chronic CSC eyes with irregular PED (13/31 eyes; 41.9%) than in those with regular PED (1/18 eyes; 5.6%)^11^. Given the fact that CSC is included in the pachychoroid spectrum disease, another study using OCTA was similar to the previous one and it revealed that type 1 neovascular tissue was visualized in most of the study eyes (21/22 eyes; 95%) with pachychoroid spectrum diagnoses and shallow irregular PED^14^. In the study of Dansingani KK *et al*.^18^, pachychoroid with neovascularization revealed only one SNP showing significant difference with neovascular AMD and 4 SNPs (one in *ARMS2*, three in *CFH*) showing significant difference with pachychoroid without neovascularization among 12 AMD-associated SNPs that were analyzed. Pachychoroid without neovascularization revealed 7 SNPs showing significant difference with neovascular AMD in their study. Although the study was different from our study because we excluded CSC patients with any evidence of choroidal neovascularization, it was similar to the present study in that two subgroups of pachychoroid disease were genetically different and one subgroup was relatively closer to the neovascular AMD.

Considering that CSC with irregular PED is more likely to develop into neovascular change, findings in the current study are in line with the previous studies that implied CSC resides within a spectrum of diseases with a pachychoroid-driven process including PPE, CSC, PNV, and PCV^4^. The current finding that CSC patients with irregular PED may have genetic overlap with neovascular AMD patients implies irregular PED in CSC might be the prodromal change for neovasularization. There have been some studies on genetic overlap between CSC and AMD. De Jong *et al*. showed that one SNP in *ARMS2* and three SNPs in *CFH* were significantly associated with chronic CSC among 19 AMD-associated SNPs that were analyzed^5^. Recent study revealed that the G allele of rs800292 and T allele of rs10490924 are significantly associated with the CNV development in patients with CSC^19^. Considering the fact that CSC with irregular PED is more likely to have hidden CNV, the result of the study corresponds with the findings of the present study which showed the frequency of the G allele of rs800292 or T allele of rs10490924 was higher in CSC with irregular PED than that in CSC without irregular PED (Table 2). Another studies demonstrated that a significant association between CSC and some of the SNPs in the *CFH* gene^20,21^. Miyake *et al*. investigated the genotypic difference between PNV and neovascular AMD^22^. The study revealed that genetic susceptibility of PNV patients to AMD was significantly lower than that of neovascular AMD; the frequency of the *ARMS2* rs10490924 minor allele (AMD-risky allele) was lower and the frequency of *the CFH* rs800292 minor allele (AMD-protective allele) was higher in PNV patients.

In addition to the genetic profile, patients with irregular PED were demographically different from patients without irregular PED. The proportion of female and the mean age of the patients were relatively higher in patients with irregular PED than those without irregular PED. Given the fact that CSC is more prevalent in men^23–25^ and the mean age is relatively younger, ranging 39–51 years^25,26^, the above demographic findings suggest that patients with irregular PED have different phenotype from the typical CSC patients than those without irregular PED have.

Association analysis between CSC without irregular PED and PCV revealed that significant difference for 3 SNPs (rs10490924 in *ARMS2* [*P* = 8.35 * 10^−6^; OR = 0.14], rs10737680 in *CFH* [*P* = 3.16 * 10^−3^; OR = 2.78], rs800292 in *CFH* [*P* = 4.91 * 10^−3^, OR = 2.81]). Similarly, association analysis between CSC without irregular PED and typical CNV, rs10490924 in *ARMS2*, rs10737680 in *CFH* were significantly different (*P* = 1.45 * 10^−3^, OR = 0.30; *P* = 3.65 * 10^−3^, OR = 2.87, respectively). Rs800292 in *CFH* showed marginally significant difference between the two groups (*P* = 6.12 * 10^−3^, OR = 2.68). Although some of the p-values did not reach the significant difference, the directions of the ORs were similar for the comparison between total CSC and typical CNV (or PCV) (total CSC vs. CNV; *P* = 0.014, OR = 1.97 [rs800292], *P* = 7.43 * 10^−3^, OR = 2.04 [rs10737680], *P* = 6.05 * 10^−3^, OR = 0.48 [rs10490924], total CSC vs. PCV; *P* = 5.59 * 10^−3^, OR = 2.19 [rs800292], *P* = 4.20 * 10^−3^, OR = 2.13 [rs10737680], *P* = 2.10 * 10^−5^, OR = 0.29 [rs10490924]). Overall, the association of *CFH* (rs800292, rs10737680) with total CSC or CSC without irregular PED was risky and the association of *ARMS2* (rs10490924) was protective, compared to typical CNV or PCV. The minor allele of the rs800292 was known to be protective for exudative AMD in Korean^27^ and Chinese^28^. This minor allele was also known to be risky for CSC in Japanese^20^ and Western European^5^ population. These findings can explain the risky association of the minor allele of the rs800292 for CSC compared to typical CNV or PCV in the current study. The minor allele of the rs10737680 was known to be protective for AMD in East Asian^29^ and Chinese^30^. There has been no previous study analyzing the association of rs10737680 with CSC. Hence, the risky association of the minor allele of the rs10737680 for CSC compared to typical CNV or PCV in the current study cannot be directly explained. The observation that some SNPs of the *CFH* have opposite effects in CSC compared to AMD was also revealed in the previous studies^5,20^, but the exact rationale and the mechanism are unclear. CFH is an inhibitor of the alternative complement pathway of the complement system. CFH also binds and interacts with adrenomedullin which has been shown to induce vasodilation of choroidal vascular beds, affect choroidal blood flow, and increase microvascular permeability^31–34^. The exact mechanisms for risk association of some SNPs in *CFH* with CSC are not clear, but the interaction of *CFH* with adrenomedullin may be the possible explanation of the association of *CFH* with CSC in that the main pathogenesis of the CSC is related with the choroidal vasodilation and hyperpermeability. In our study, the minor alleles of the rs800292 and rs10737680 were risky for only CSC without irregular PED, not for CSC with irregular PED, compared to typical CNV or PCV. Further studies on the exact mechanisms for the risky association of some SNPs in *CFH* only with CSC without irregular PED (and not with CSC with irregular PED) are warranted. The minor allele of the rs10490924 was known to be risky for exudative AMD in Korean^27^ and for AMD in East Asian^29^. This minor allele was also reported to be protective for CSC in Western European^5^. These findings can explain the protective association of the minor allele of the rs10490924 for CSC compared to typical CNV or PCV in the current study. A recent study revealed that ARMS2 interacts with components of the extracellular matrix (ECM)^35^. Disturbances in the ECM at the level of RPE and/or choroid could affect the susceptibility to cellular detachments, which may be the possible mechanisms for the protective association of *ARMS2* with CSC^5^.

**Figure 2:**
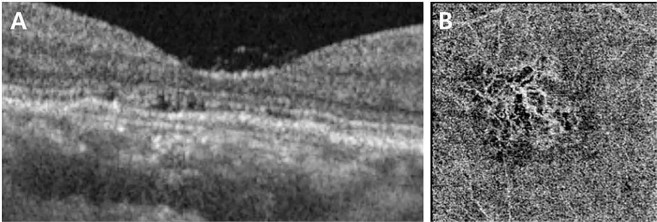
Representative case with irregular pigment epithelial detachment (PED) which showed choroidal neovascularization (CNV) in optical coherence tomography angiography (OCTA). (**A**) The OCTA B-scan image showed irregular PED in the right eye. (**B**) The OCTA of the same patient showed CNV in choriocapillaris layer.

Our study has some limitations. First is its cross-sectional study design, which may have resulted in inherent limitations. Second, we analyzed only 10 known AMD-associated SNPs and could not cover whole genome. Considering that genetic association may be affected with ethnicity, we analyzed limited number of main SNPs based on previous studies^27,29^ showing the replicated association with AMD in East Asian or Korean. Third, OCTA was performed only in small portion of the CSC patients with irregular PED (Fig. 2). To reveal the association between CSC with irregular PED and CNV (or PCV), further long term follow up study with more OCTA data of CSC patients with irregular PED is needed. Finally, there has been no consensus or established definition of irregular PED. We newly defined the ‘irregular PED’ as a flat, irregular non-dome-shaped protrusion of RPE with ‘at least partially hyper-reflective’ sub-RPE fluid and analyze the genetic difference between CSC patients with irregular PED and those without irregular PED.

Among 10 known AMD-associated SNPs, CSC without irregular PED showed significant difference with typical CNV in 2 SNPs and with PCV in 3 SNPs. In contrast, CSC with irregular PED revealed no SNP showing significant difference compared with typical CNV, and only one SNP showing significant difference with PCV. In the association analysis between CSC with irregular PED and CSC without irregular PED, rs800292 in *CFH* were significantly different, and rs10737680 in *CFH* showed marginally significant difference. In conclusion, these findings suggest patients with irregular PED are genetically different from those without irregular PED and may have genetic and pathophysiologic overlap with AMD patients.

## Methods

The present study is a cross-sectional study. This study was approved by the Institutional Review Board (IRB) of Seoul National University Bundang Hospital (SNUBH; IRB No.B-1105/127-014, Seongnam, South Korea) and adhered to the tenets of the Declaration of Helsinki. Written informed consent was obtained from all subjects before participation in the study.

### Study participant

Patients with CSC, exudative AMD with typical CNV, and PCV were recruited at SNUBH retina clinic from January 2009 to January 2016. All patients underwent comprehensive ophthalmologic evaluation, including measurement of best-corrected visual acuity, slit-lamp biomicroscopy, indirect fundus exam, fluorescein angiography (FA), indocyanine green angiography (ICGA, Heidelberg Retina Angiography; Heidelberg Engineering, Heidelberg, Germany), and optical coherence tomography (OCT, Spectralis OCT; Heidelberg Engineering, Heidelberg, Germany).

The CSC group was defined as a group of patients with following characteristics: serous subretinal fluid on optical coherence tomography, focal leakage spot (ink blot) or smokestack pattern or ≥1 area of multifocal diffuse leakage, or presence of irregular retinal pigment epithelium window defects on fluorescein angiography and corresponding hyperfluorescence on indocyanine green angiography^36–38^. Patients receiving PDT before the first spectral-domain (SD) OCT at our institute or patients receiving exogenous corticosteroids were excluded. Patients showing evidence of diabetic retinopathy, retinal vascular diseases, or other diseases that can cause macular exudation, such as exudative age-related macular degeneration (AMD), PCV were excluded in the CSC patients.

The typical CNV group was determined by demonstration of choroidal neovascular membrane on FA without any evidence of PCV on ICGA. According to the recent studies suggesting PCV belongs to pachychoroid spectrum diseases^4,39,40^, we analyzed the typical CNV and PCV patients separately. Cases having evidence of retinal–retinal or retinal–choroidal anastomosis on ICGA were defined as retinal angiomatous proliferation (RAP). Patients with RAP having choroidal neovascular membrane were included in the typical CNV group. The PCV group was defined as hyperfluorescent polypoidal choroidal vasculature with branching vascular network on ICGA with concomitant exudation or hemorrhage^41^.

### Definition of Irregular PED and subgroups of CSC

**Figure 3:**
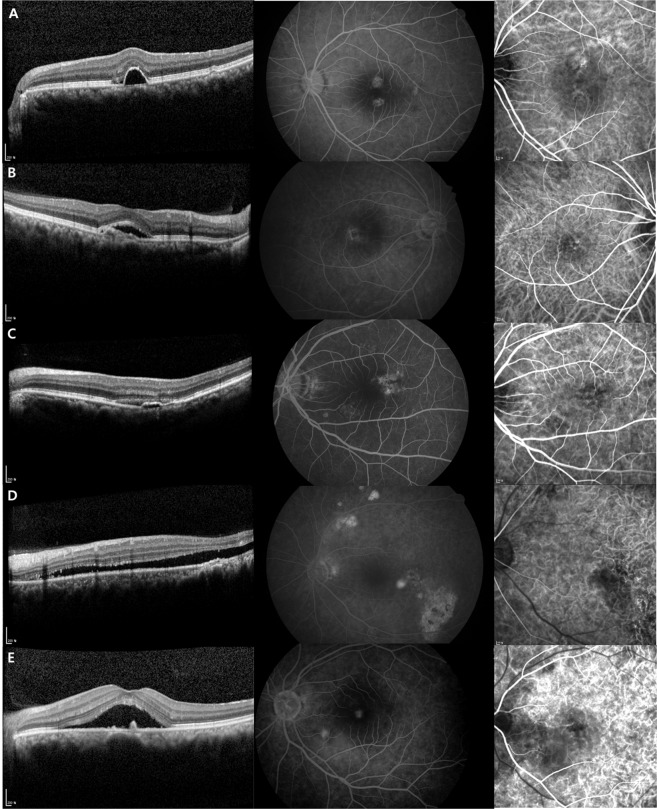
Representative optical coherence tomography images, corresponding fluorescein angiography (FA) images, and indocyanine green angiography (ICGA) images of regular pigment epithelial detachment (PED), flat PEDs, and retinal pigment epithelium (RPE) bump. (**A**) Case with regular PED. A dome-shaped protrusion of RPE with sub-RPE fluid was seen. FA showed parafoveal multifocal leakages. ICGA showed enlarged choroidal vasculatures and choroidal hyperpermeability. (**B**) Case with irregular PED. A flat, non-dome-shaped protrusion of RPE with at least partially hyper-reflective sub-RPE fluid was defined as an ‘irregular PED’. (Optical density of the sub-RPE fluid could be evaluated only when the height of the sub-RPE fluid ≥ 20 *μ*m). FA showed parafoveal multiple leakages. ICGA showed enlarged choroidal vasculatures. (**C**) Case with flat PED with hypo-reflective sub-RPE fluid. A flat, non-dome-shaped protrusion of RPE with ‘not optically filled‘ sub-RPE fluid was seen (height of sub-RPE fluid ≥ 20 um). FA showed multiple parafoveal and perifoveal leaking points. ICGA showed enlarged choroidal vasculatures and hyperpermeability. (**D**) Case with flat PED with indeterminate optical density of sub-RPE fluid. A flat, non-dome-shaped protrusion of RPE with low height of sub-RPE fluid that is hard to evaluate the optical density of the sub-RPE fluid was seen (height of sub-RPE fluid < 20 um). FA showed temporal perifoveal late leakage (ink blot pattern). ICGA showed choroidal hyperpermeability. (**E**) Case with RPE bump. Small irregular protrusion of RPE without sub-RPE fluid was seen. FA showed foveal and perifoveal leaking points. ICGA showed choroidal hyperpermeability.

Based on the OCT findings in patients with CSC^42^, a dome-shaped protrusion of RPE with sub-RPE fluid was defined as a ‘regular PED’. A flat, irregular non-dome-shaped protrusion of RPE with ‘at least partially hyper-reflective’ sub-RPE fluid was defined as an’irregular PED’. According to the optical density of the sub-RPE fluid of flat PED, there were also categories of flat PED with ‘hypo-reflective’ sub-RPE fluid and flat PED with ‘indeterminate optical density’ (i.e., flat PED with <20 *μ*m height of the sub-RPE fluid; optical density of the sub-RPE fluid could be evaluated only when the height of the sub-RPE fluid ≥20 *μ*m.) Small irregular protrusion of RPE without sub-RPE fluid was defined as a ‘RPE bump’. Using high speed mode with the spectralis OCT, we checked the 30° * 20° field of view (8.7 mm * 5.8 mm) obtained from the 25 serial B scan to classify the PED. Representative OCT images of regular PED, flat PEDs, and RPE bump were shown in Fig. 3. CSC patients were divided into two subgroups; (1) CSC with ‘irregular PED’, (2) CSC without ‘irregular PED’. CSC without ‘irregular PED’ included cases with ‘regular PED’, flat PED with ‘hypo-reflective’ sub-RPE fluid, or with ‘indeterminate optical density’, RPE bump, or only sub-retinal fluid (SRF).

Finally, study participants were classified into five subgroups; (1) total CSC (i.e., CSC with or without ‘irregular PED’) (2) CSC with ‘irregular PED’ (3) CSC without ‘irregular PED’ (4) typical CNV and (5) PCV.

### Genotype analysis

DNA was extracted from whole blood by DNA extraction kit (QIAamp DNA Maxi kit, Qiagen Inc.). Study samples processed on the HumanExome BeadChip (Illumina, Inc., San Diego, CA) includes 243,345 markers focused on protein-altering variants. Details about SNP content and selection strategies can be found at the exome array design webpage (see http://genome.sph.umich.edu/wiki/Exome_Chip_Design). Genotype calling was carried out using Illumina’s GenTrain version 2.0 clustering algorithm in Genome Studio software (V2011.1). Cluster boundaries were determined using Illumina’s standard cluster file. After visual inspection on SNPs with high missing rates, completely missing SNPs (call rate = 0) were removed for further analysis. In total, 646 samples [CSC (n = 281), CNV (n = 221), PCV (n = 144) were successfully genotyped with average call rate >99%.

### Statistical analysis

Demographics of the study population were compared among subgroups using the t-test, χ2 test and the one-way analysis of variance (ANOVA). Genotype data cleaning and analysis was performed using PLINK (http://pngu.mgh.harvard.edu/purcell/plink). Among the genotyped SNPs, twelve major AMD-associated SNPs including 9 SNPs replicated in the previous studies of East Asian^29^, and 2 SNPs replicated in the previous studies of Korean^27^ were analyzed to investigate the difference of genetic profile and association with AMD among subgroups. The SNP data was cleaned by removing SNPs with a low genotyping pass rate (greater than 5% of genotypes missing from the entire cohort), SNPs with a low minor allele frequency (less than 0.2% of MAF), and SNPs in those subjects without disease that were not in Hardy Weinberg Equilibrium (HWE p < 10^−6^). After quality control, among 12 major AMD-associated SNPs, 10 SNPs (including 8 SNPs replicated in the previous studies of East Asian, and 1 SNP replicated in the previous study of Korean) remained for further analysis. The remained 10 SNPs were rs800292, rs1061170, rs10737680 in *CFH*, rs6795735 in *ADAMTS9*, rs4698775 in *CFI*, rs429608 in *C2-CFB*, rs943080 in *VEGFA*, rs334353 in *TGFBR1*, rs10490924 in *ARMS2-HTRA1*, and rs3764261 in *CETP*. After quality control, 280 patients with CSC, 203 patients with CNV, and 135 patients with PCV remained for the statistical analysis.

The OCT-based phenotyping for CSC was performed independently by two observers (S.C.C and N-K.R); 1) regular PED, 2) flat PED with ‘at least partially hyper-reflective’ sub-RPE fluid (=i.e., irregular PED), 3) flat PED with ‘hypo-reflective’ sub-RPE fluid, 4) flat PED with ‘indeterminate optical density’, 5) RPE bump, and 6) only SRF. All measurements were performed based on 1:1 micron images. The inter-observer agreement of the phenotyping was evaluated with Cohen’s kappa (*k* = 0.916). When discrepancies arose for the OCT-based phenotyping, the two observers discussed their evaluations and came to an agreement for final phenotype.

The analysis of 10 SNPs was performed in each pair of subgroups: (1) between CSC with ‘irregular PED’ and CSC without ‘irregular PED’; (2) between total CSC with and typical CNV (or PCV); (3) between CSC with ‘irregular PED’ and typical CNV (or PCV); and (4) between CSC without ‘irregular PED’ and typical CNV (or PCV). For the SNP analysis, the minor allele, or less frequent allele, for each SNP was tested for association with disease of the case for each pair of the subgroups mentioned above using the chi square test in PLINK. Logistic regression was also performed to investigate the age, sex adjusted association of the genotype with disease of the case. A Bonferroni correction was performed, and the individual test for the 10 SNPs were considered significant at P < 0.005, corrected for 10 tests. The p-value of the age, sex adjusted analysis using logistic regression was finally used to determine the significant association.

## Supplementary information

supplementary information.

## Data Availability

The datasets generated and/or analyzed during the current study are available from the corresponding author on reasonable request.
