# Supplementary material for: Association of Irregular Pigment Epithelial Detachment in Central Serous Chorioretinopathy with Genetic Variants Implicated in Age-related Macular Degeneration
Source: Sci Rep. 2020 Jan 27;10:1203. doi: 10.1038/s41598-020-57747-8 (PMC6985219; doi:10.1038/s41598-020-57747-8)
Supplement: Supplementary file 1 — supplementary information. [file 41598_2020_57747_MOESM1_ESM.docx]

**Association of Irregular Pigment Epithelial Detachment in Central Serous Chorioretinopathy with Genetic Variants Implicated in Age-related Macular Degeneration**

Name of authors:

Soo Chang Cho, MD, MS^1, 2^, Na-Kyung Ryoo, MD, MS^1, 3^, Jeeyun Ahn, MD, PhD^4^, Se Joon Woo, MD, PhD^1^, Kyu Hyung Park, MD, PhD^1^*

Institutional affiliation:

^1^Department of Ophthalmology, Seoul National University College of Medicine, Seoul National University Bundang Hospital, Seongnam, Republic of Korea

^2^Department of Ophthalmology, Ewha Womans University Mokdong Hospital, Seoul, Republic of Korea

^3^Department of Ophthalmology, Veterans Health Service Medical Center, Seoul, Republic of Korea

^4^Department of Ophthalmology, Seoul National University College of Medicine, Seoul Metropolitan Government Seoul National University Boramae Medical Center, Republic of Korea

Address correspondence and reprint requests to

* Kyu Hyung Park, MD, PhD

Department of Ophthalmology, Seoul National University Bundang Hospital, 173-82 Gumi-ro, Bundang-gu, Seongnam-si, Gyeonggi-do, 13620, Republic of Korea

Tel: +82-31-787-7373 Fax: +82-31-787-4057

E-mail: jiani4@snu.ac.kr

**Supplementary Table 1. Analysis of 10 Age-Related Macular Degeneration Loci in ‘Central Serous Chorioretinopathy’ and ‘Typical Choroidal Neovascularization’ group.**

| **Case** | **Control** | **SNP** | **Gene** | **Allele** | **Minor Allele Frequency** | |  | **Allelic association** | |  | **Age, sex adjusted analysis** | |
| --- | --- | --- | --- | --- | --- | --- | --- | --- | --- | --- | --- | --- |
|  |  |  |  |  | case | control |  | p-value | Odds Ratio (95% CI) |  | p-value | Odds Ratio (95% CI) |
| Total CSC (N=280) | CNV  (N=203) | rs800292 | *CFH* | G>A | 0.4643 | 0.2734 |  | **1.73E-09** | 2.303 (1.751-3.030) |  | 0.01393 | 1.969 (1.147-3.378) |
|  |  | rs1061170 | *CFH* | T>C | 0.08393 | 0.1238 |  | 0.04251 | 0.649 (0.426-0.988) |  | 0.1343 | 0.528 (0.229-1.218) |
|  |  | rs10737680 | *CFH* | A>C | 0.5 | 0.33 |  | **1.55E-07** | 2.030 (1.556-2.650) |  | *0.007428* | 2.036 (1.210-3.426) |
|  |  | rs6795735 | *ADAMTS9* | T>C | 0.1857 | 0.1946 |  | 0.7285 | 0.944 (0.682-1.307) |  | 0.8846 | 1.045 (0.576-1.898) |
|  |  | rs4698775 | *CFI* | T>G | 0.2036 | 0.2069 |  | 0.8994 | 0.980 (0.714-1.344) |  | 0.2157 | 0.667 (0.352-1.266) |
|  |  | rs429608 | *C2-CFB* | G>A | 0.0875 | 0.06404 |  | 0.1786 | 1.401 (0.855-2.296) |  | 0.6832 | 1.217 (0.475-3.118) |
|  |  | rs943080 | *VEGFA* | T>C | 0.3054 | 0.2602 |  | 0.1295 | 1.250 (0.937-1.668) |  | 0.06644 | 1.772 (0.962-3.263) |
|  |  | rs334353 | *TGFBR1* | T>G | 0.4679 | 0.453 |  | 0.6473 | 1.062 (0.821-1.373) |  | 0.1 | 1.552 (0.919-2.621) |
|  |  | rs10490924 | *ARMS2-HTRA1* | G>T | 0.3643 | 0.6725 |  | **4.64E-21** | 0.279 (0.213-0.366) |  | *0.006049* | 0.477 (0.281-0.809) |
|  |  | rs3764261 | *CETP* | G>T | 0.1679 | 0.1626 |  | 0.827 | 1.039 (0.736-1.466) |  | 0.494 | 0.779 (0.381-1.593) |

Bold character indicates statistically significance (P < 0.005, Bonferroni correction).

Italic character indicates marginal significance.

**Supplementary Table 2. Analysis of 10 Age-Related Macular Degeneration Loci in ‘Central Serous Chorioretinopathy’ and ‘Polypoidal Choroidal Vasculopathy’ group.**

| **Case** | **Control** | **SNP** | **Gene** | **Allele** | **Minor Allele Freqeuncy** | |  | **Allelic association** | |  | **Age, sex adjusted analysis** | |
| --- | --- | --- | --- | --- | --- | --- | --- | --- | --- | --- | --- | --- |
|  |  |  |  |  | case | control |  | p-value | Odds Ratio (95% CI) |  | p-value | Odds Ratio (95% CI) |
| Total CSC (N=280) | PCV  (N=135) | rs800292 | *CFH* | G>A | 0.4643 | 0.2926 |  | **2.42E-06** | 2.095 (1.536-2.858) |  | *0.005587* | 2.189 (1.258-3.808) |
|  |  | rs1061170 | *CFH* | T>C | 0.08393 | 0.1037 |  | 0.3519 | 0.792 (0.484-1.295) |  | 0.2137 | 0.598 (0.266-1.345) |
|  |  | rs10737680 | *CFH* | A>C | 0.5 | 0.3321 |  | **5.41E-06** | 2.011 (1.485-2.724) |  | **0.004195** | 2.131 (1.270-3.578) |
|  |  | rs6795735 | *ADAMTS9* | T>C | 0.1857 | 0.2074 |  | 0.4579 | 0.872 (0.606-1.253) |  | 0.9514 | 1.019 (0.560-1.852) |
|  |  | rs4698775 | *CFI* | T>G | 0.2036 | 0.2111 |  | 0.8013 | 0.955 (0.668-1.366) |  | 0.4616 | 0.795 (0.431-1.465) |
|  |  | rs429608 | *C2-CFB* | G>A | 0.0875 | 0.06296 |  | 0.2209 | 1.427 (0.806-2.528) |  | 0.337 | 1.574 (0.623-3.976) |
|  |  | rs943080 | *VEGFA* | T>C | 0.3054 | 0.312 |  | 0.846 | 0.969 (0.707-1.329) |  | 0.8146 | 1.068 (0.616-1.851) |
|  |  | rs334353 | *TGFBR1* | T>G | 0.4679 | 0.3977 |  | 0.05875 | 1.331 (0.989-1.792) |  | 0.279 | 1.309 (0.804-2.133) |
|  |  | rs10490924 | *ARMS2-HTRA1* | G>T | 0.3643 | 0.6493 |  | **1.34E-14** | 0.310 (0.228-0.420) |  | **2.10E-05** | 0.290 (0.164-0.513) |
|  |  | rs3764261 | *CETP* | G>T | 0.1679 | 0.2111 |  | 0.1302 | 0.754 (0.522-1.088) |  | 0.007805 | 0.403 (0.206-0.787) |

Bold character indicates statistically significance (P < 0.005, Bonferroni correction).

Italic character indicates marginal significance.

**Supplementary Table 3. Analysis of 10 Age-Related Macular Degeneration Loci in ‘Irregular Pigment Epithelial Detachment (+) of Central Serous Chorioretinopathy’ and ‘Polypoidal Choroidal Vasculopathy’ group.**

| **Case** | **Control** | **SNP** | **Gene** | **Allele** | **Minor Allele Freqeuncy** | |  | **Allelic association** | | |  | **Age, sex adjusted analysis** | |
| --- | --- | --- | --- | --- | --- | --- | --- | --- | --- | --- | --- | --- | --- |
|  |  |  |  |  | case | control |  | p-value | | Odds Ratio (95% CI) |  | p-value | Odds Ratio (95% CI) |
| Irregular PED (+)  in CSC (N=126) | PCV  (N=135) | rs800292 | *CFH* | G>A | 0.3889 | 0.2926 |  | 0.02021 | 1.539 (1.069-2.215) | |  | 0.1261 | 1.637 (0.871-3.077) |
|  |  | rs1061170 | *CFH* | T>C | 0.1071 | 0.1037 |  | 0.8982 | 1.037 (0.593-1.814) | |  | 0.5849 | 0.787 (0.334-1.857) |
|  |  | rs10737680 | *CFH* | A>C | 0.4365 | 0.3321 |  | 0.01435 | 1.558 (1.092-2.224) | |  | 0.09932 | 1.626 (0.912-2.900) |
|  |  | rs6795735 | *ADAMTS9* | T>C | 0.2183 | 0.2074 |  | 0.7622 | 1.067 (0.701-1.623) | |  | 0.8856 | 0.952 (0.487-1.862) |
|  |  | rs4698775 | *CFI* | T>G | 0.2302 | 0.2111 |  | 0.5998 | 1.117 (0.738-1.690) | |  | 0.4064 | 0.752 (0.383-1.475) |
|  |  | rs429608 | *C2-CFB* | G>A | 0.06349 | 0.06296 |  | 0.9802 | 1.009 (0.498-2.043) | |  | 0.9614 | 0.971 (0.297-3.174) |
|  |  | rs943080 | *VEGFA* | T>C | 0.2778 | 0.312 |  | 0.3931 | 0.848 (0.581-1.238) | |  | 0.9858 | 1.006 (0.536-1.887) |
|  |  | rs334353 | *TGFBR1* | T>G | 0.4802 | 0.3977 |  | 0.05922 | 1.399 (0.987-1.983) | |  | 0.3811 | 1.296 (0.725-2.318) |
|  |  | rs10490924 | *ARMS2-HTRA1* | G>T | 0.4167 | 0.6493 |  | **1.06E-07** | 0.386 (0.271-0.550) | |  | **0.004167** | 0.411 (0.223-0.755) |
|  |  | rs3764261 | *CETP* | G>T | 0.1746 | 0.2111 |  | 0.2914 | 0.791 (0.511-1.224) | |  | 0.01732 | 0.400 (0.188-0.851) |

Bold character indicates statistically significance (P < 0.005, Bonferroni correction).

Italic character indicates marginal significance.

**Supplementary Table 4. Analysis of 10 Age-Related Macular Degeneration Loci in ‘Irregular Pigment Epithelial Detachment (-) of Central Serous Chorioretinopathy’ and ‘Polypoidal Choroidal Vasculopathy’ group.**

| **Case** | **Control** | **SNP** | **Gene** | **Allele** | **Minor Allele Frequency** | |  | **Allelic association** | |  | **Age, sex adjusted analysis** | |
| --- | --- | --- | --- | --- | --- | --- | --- | --- | --- | --- | --- | --- |
|  |  |  |  |  | case | control |  | p-value | Odds Ratio (95% CI) |  | p-value | Odds Ratio (95% CI) |
| Irregular PED (-)  in CSC (N=154) | PCV  (N=135) | rs800292 | *CFH* | G>A | 0.526 | 0.2926 |  | **1.37E-08** | 2.683 (1.901-3.786) |  | **0.004906** | 2.812 (1.368-5.779) |
|  |  | rs1061170 | *CFH* | T>C | 0.06494 | 0.1037 |  | 0.09196 | 0.600 (0.330-1.092) |  | 0.06553 | 0.315 (0.092-1.077) |
|  |  | rs10737680 | *CFH* | A>C | 0.5519 | 0.3321 |  | **1.22E-07** | 2.478 (1.765-3.478) |  | **0.003158** | 2.782 (1.410-5.489) |
|  |  | rs6795735 | *ADAMTS9* | T>C | 0.1591 | 0.2074 |  | 0.1328 | 0.723 (0.473-1.105) |  | 0.5138 | 1.306 (0.586-2.910) |
|  |  | rs4698775 | *CFI* | T>G | 0.1818 | 0.2111 |  | 0.3756 | 0.830 (0.550-1.253) |  | 0.946 | 0.973 (0.435-2.174) |
|  |  | rs429608 | *C2-CFB* | G>A | 0.1071 | 0.06296 |  | 0.05941 | 1.786 (0.971-3.285) |  | 0.1147 | 2.388 (0.810-7.042) |
|  |  | rs943080 | *VEGFA* | T>C | 0.3279 | 0.312 |  | 0.6841 | 1.076 (0.757-1.530) |  | 0.5039 | 1.264 (0.636-2.513) |
|  |  | rs334353 | *TGFBR1* | T>G | 0.4578 | 0.3977 |  | 0.148 | 1.279 (0.916-1.784) |  | 0.4473 | 1.271 (0.684-2.362) |
|  |  | rs10490924 | *ARMS2-HTRA1* | G>T | 0.3214 | 0.6493 |  | **3.86E-15** | 0.256 (0.181-0.362) |  | **8.35E-06** | 0.142 (0.060-0.336) |
|  |  | rs3764261 | *CETP* | G>T | 0.1623 | 0.2111 |  | 0.132 | 0.724 (0.475-1.103) |  | 0.06281 | 0.429 (0.176-1.046) |

Bold character indicates statistically significance (P < 0.005, Bonferroni correction).

Italic character indicates marginal significance.
